# Scanning Electron Microscopy of Conjunctival Scraping: Our Experience in the Diagnosis of Infectious Keratitis with Negative Culture Tests

**DOI:** 10.3390/reports6010010

**Published:** 2023-02-27

**Authors:** Mario Troisi, Salvatore Del Prete, Salvatore Troisi, Daniela Marasco, Ciro Costagliola

**Affiliations:** 1 Department of Neurosciences, Reproductive and Dentistry Sciences, University of Naples Federico II, 80131 Naples, Italy; 2 Service Biotech s.r.l., 80121 Naples, Italy; 3 Department of Head-Neck, Ophthalmologic Unit, Salerno Hospital University, 84100 Salerno, Italy

**Keywords:** infectious keratitis, scanning electron microscopy diagnosis, ocular surface infections, correlative microscopy, microbial keratitis, conjunctival SEM examination, conjunctival diagnostic scraping, corneal ulcer diagnosis

## Abstract

65 consecutive patients with presumed microbial keratitis and negative culture tests for bacteria and fungi obtained by corneal curettage were evaluated. All patients had undergone local broad spectrum antibiotic therapy for at least 5 days with no clinical improvement. After 48–72 h of wash-out they underwent scraping of the superior tarsal conjunctiva for cytological examination of cellular morphology in Scanning Electron Microscopy (SEM). The presence of pathogenic microorganisms was detected with this method in 62 of these patients, towards which specific therapy was carried out. Clinical improvement and eradication of microorganisms previously detected by SEM examination were observed in all positive patients over a time between 10 and 49 days. In three patients, no microorganisms were detected, but the presence of inflammatory cells (eosinophils and mast cells) or dry eye findings. This method could be useful to detect the presence of non-isolated microorganisms at common culture tests. The resolution of the infectious keratitis and the eradication of the pathogens at the subsequent cytological examination of cellular morphology in Scanning Electron Microscopy support the validity of the proposed method.

## 1. Introduction

Keratitis can result from infections, allergies or toxic agents. Lesions of the epithelium or even of the corneal stroma can also be present in dry eye, in neurotrophic forms and incomplete closure of the eye; the clinical characteristics and the inflammatory process generally point towards infectious or other forms [1]. A bacterial origin indicative of acute infectious conjunctivitis can be established with a greater or lesser probability from the answers to simple questions asked during the anamnesis: the presence of sticky eyes in the early morning increases the probability of a bacterial cause, while itching and history of conjunctivitis decreases it and directs towards allergic forms or eyelid inflammation [2]. Pseudomonas keratitis diagnosis is made significantly easier by larger infiltrate finding; for Acanthamoeba keratitis when observing a ring infiltrate [1]. However, the history and physical examination are often insufficient for a correct clinical diagnosis, for which laboratory tests can play a vital role in identifying specific pathogens: different types of inflammations can often have similar clinical signs and the use of antimicrobials before ophthalmological examination reduces the chances of a correct microbiological diagnosis and hinders its identification [3]. In many of these cases, prior antibiotic therapy or low bioburden on the scraped surface or inadequate sampling can lead to a false negative culture test result [4]. In other cases, the causative organism is not evident using common culture media, as in Acanthamoeba infections [5]. This paper presents the results of superior tarsal conjunctiva scrapings examined by Scanning Electron Microscopy (SEM) in patients with suspected infectious keratitis who tested negative on common culture tests and with an unsatisfactory response to empiric antimicrobial therapies. SEM is used to examine three-dimensional surfaces, whereas transmission EM (TEM), developed before SEM, is used to probe the structures of fixed, sectioned or freeze-etched fungal samples, producing two-dimensional (flat) images. These images can be combined, at first laboriously, and now computationally, to provide detailed information about cytoplasmic organization [6]. It consists of a sample analysis method widely used in research and clinical diagnostics since the 80s, when the first applications of the method for diagnostic purposes were described [7,8]. In particular, this technique has been successfully used in the identification of bacteria and to analyze the structural organization of parasitic protozoa and their interaction with host cells [9,10]. In addition, impression cytology with scanning electron microscopy (SEM) was used to study the ocular surface of human eyes, detecting a reduction or absence of microvilli in patients affected by tear film abnormalities [11].

In the present study the ability of SEM to detect pathogenic microorganisms from upper tarsal conjunctiva scraping in patients affected by suspected infectious keratoconjunctivitis with negative culture test for bacteria and fungi was evaluated. The criteria to establish the efficacy of this method were the clinical response to the prescribed treatment following the results of the cytological examination of the cell morphology in Scanning Electron Microscopy and the eradication of the microorganisms at the subsequent control examination. 

## 2. Materials and Methods

### 2.1. Samples Collection 

65 eyes of 65 consecutive patients presenting signs and symptoms consistent with microbial keratitis, but with negative bacterial and fungal sequencing tests, were examined. All patients had undergone broad-spectrum local antibiotic therapy for at least 5 days without any clinical improvement. Patients with ulcerations deeper than 80% of the corneal thickness, age < 18 years, pregnant or lactating women were excluded. For each suspected case of infectious keratitis, information relating to clinical data, duration of symptoms, risk factors and occupational status were documented according to a detailed protocol. The ophthalmological evaluation was performed by a corneal specialist using a slit lamp biomicroscope and the results were recorded in a predefined format. Detailed schematic documentation of the ulcer was recorded at first observation and at follow-up. After at least 48 h of washout, conjunctival scrapings of the superior tarsal conjunctiva with a Kimura spatula were performed and subjected to cytological evaluation by scanning electron microscopy (SEM), as described below. In case of bilateral keratitis, the eye with the most severe signs of inflammation and corneal damage was examined with this method; when pathogens were detected, the other eye was also treated with the same therapy. In presence of corneal dendritic ulcers or a history of a previous herpes infection in the same eye, the herpes virus rapid test was also performed. The treatment was set according to the clinical data and the results of the electron microscope examination. Patients were monitored every 2–7 days, depending on the severity of clinical manifestations, by slit-lamp examination and vital staining. Once a significant clinical improvement was obtained or in presence of worsening of the corneal conditions and inflammation, the cytological examination of the cell morphology in Scanning Electron Microscopy was repeated, after three days of washout. In case of further detection of pathogenic microorganisms, the therapy has been remodeled on the basis of the results of the cytological examination of the cell morphology in Scanning Electron Microscopy. Treatment regimen, treatment response, and final outcome were recorded in all cases.

### 2.2. Scanning Electron Microscopy Examination

Cytological Method of examination in SEM: the cytological sampling by scraping technique of the tarsal conjunctiva with a smooth spatula were performed [11,12]. The conjunctiva mucosal cells and all secretions were placed on slide (Super Frost Plus Menzel-Gläser, Thermo Scientific, Milan, Italy). The cells were then stained according to the panoptic method (3 min in pure May-Grunwald dye [Carlo Erba, Milan, Italy], 6 min in 50% May-Grunwald dye; 1 min in bidistilled water [Carlo Erba, Milan, Italy]; and 30 min in Giemsa solution [Carlo Erba, Milan, Italy] diluted 1:10 *v*/*v*). The slide was then covered with a glass cover with dimensions of 24 × 50 mm and observed under an optical microscope (Nikon Eclipse 50i) at 100 × oil-immersion enlargement. The images were recorded using a Nikon DS1 camera and digitized using a NIS-D elements computer support. SEM method, applied to the practice of scraping cytology of the tarsal conjunctiva, was carried out by positioning the mucosal secretion on a 13 mm DIA round slide (Agar scientific). The round slide sample was fixed in 2% gluteraldehyde, then washed in PBS at 7.4 pH for 15 min for 3 times; then treated in OSMIO 4% for 2 h. Then the samples were washed twice in PBS at pH 7.4 [Carlo Erba, Milan, Italy], 30 min each time; finally the samples thus treated were dehydrated in alcohol at increasing concentrations: 30% at 25 min, 50% at 25 min, 70% at 20min and 96% at 20 min for two times. Once dehydrated, they were placed in critical-point CO_2_ (critical point at 31 °C and 73 atm) (Leica EM CPD300). The preparation was viewed in scanning microscopy with JEOL microscope supplied by University of Naples Federico II [13,14]. The samples treatment allowed to visualize at various magnifications the bacterial species colonizing the ocular mucosa and the typical inflammatory cells; in addition, attention was paid to all pathogens involved in the ocular mucosa phlogosis of the patient under examination. The use of correlative microscopy put in evidence the correlation between phlogistic cells (evidenced with classical cytology most) and pathogens identified with scanning electron microscopy; this technique facilitates the diagnosis and the therapeutic treatment [15,16].

## 3. Results

### Patients Clinical Data

**Figure 1:**
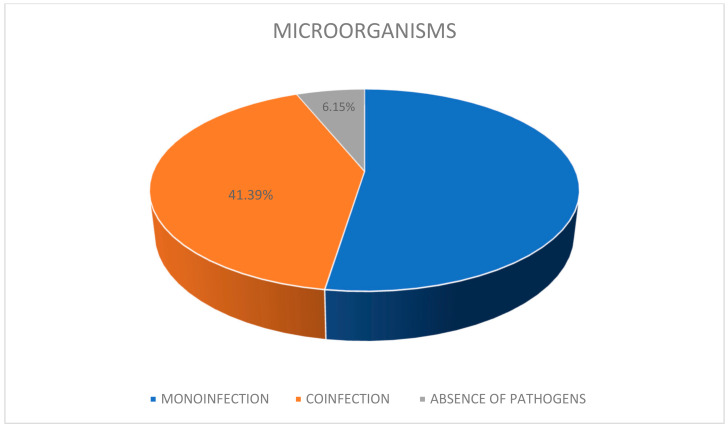
SEM examination: Presence of microorganisms in scraping of the superior tarsal conjunctiva.

65 eyes of 65 patients, 30 male and 35 female, were examined. Average age 53.4 years (range 15–86). 41 patients were affected by systemic diseases for which they underwent oral or parenteral therapies; the anamnestic examination did not reveal relevant systemic pathologies in the other 24 patients. Cytological examination of cell morphology in Scanning Electron Microscopy (SEM) allowed the identification of pathogenic microorganisms in 61 eyes; in 32 (52.46%) of them two or more pathogens were present (Figure 1).

In the four patients in whom the presence of pathogenic microorganisms was not detected, microscopic signs of dry eyes or allergic reactions were found. The age and gender of each patient, the ophthalmological diagnosis, the presence of associated systemic pathologies, the microorganisms identified, the topical antimicrobial therapies performed and the resolution times of the infectious process are summarized in Table 1. 

**Figure 2:**
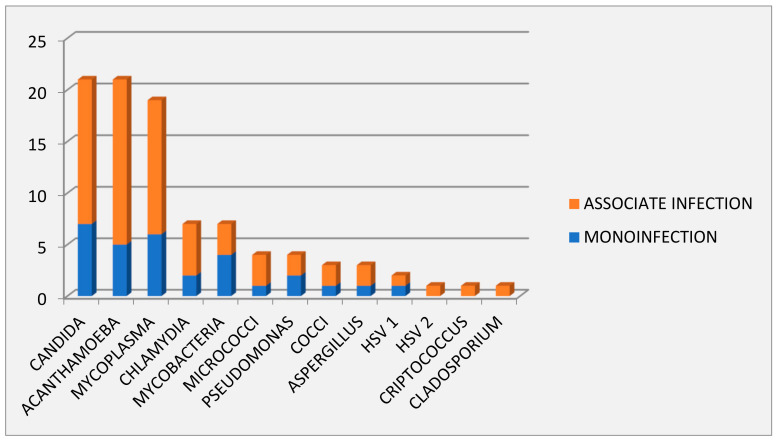
Evaluation by scanning electron microscopy: single organism infections and polyinfections.

The pathogens found are, in order of frequency: Candida (21), Acanthamoeba (21), Mycoplasma (19), Chlamydia (7), Mycobacteria (7), Micrococci (4), Pseudomonas (4), Cocci (3), Aspergillus (3), HSV1 (2), HSV2 (1), Cryptococcus (1), Cladosporium (1); in most cases the detection of Candida, Acanthamoeba, Mycoplasma, Chlamydia and Micrococci was associated with other microorganisms (Figure 2).

The negative result of the cytological examination of the cell morphology in the SEM report and a significant clinical improvement were obtained in a period of time ranging from 14 to 49 days (mean 26.6 days). In 19 cases of positive patients it was necessary to repeat the examination performed after improvement of the clinical conditions as the therapeutic regime set had not been sufficient to eradicate the microorganisms found. The therapeutic scheme was modified on the basis of the results obtained and the scraping was repeated for the SEM examination after a new treatment cycle and the observation of a marked improvement in the clinical picture. Intolerance to the prescribed drugs was highlighted in 5 patients, such as to require the replacement of the products used.

## 4. Discussion

**Figure 3:**
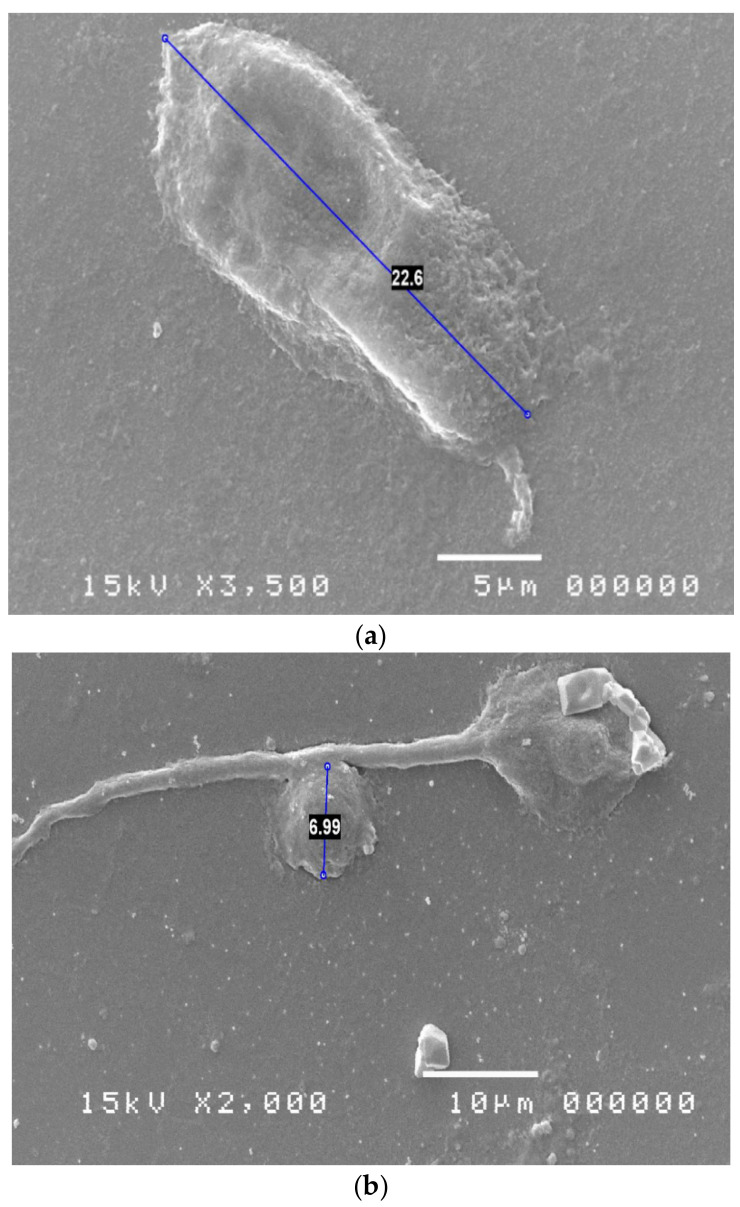

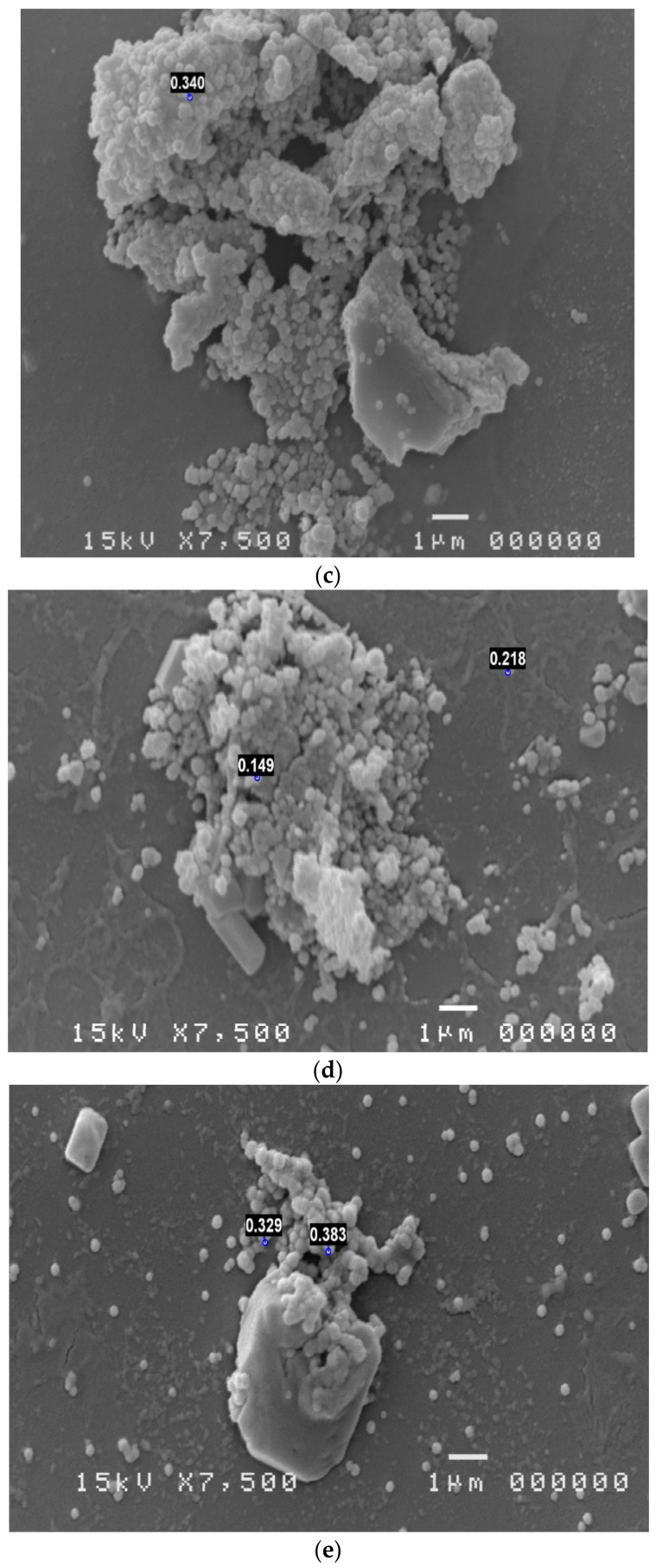
SEM Evaluation: Acanthamoeba (**a**), Candida (**b**), Chlamydia (**c**), Mycobacteria (**d**), Mycoplasmas (**e**).

Infectious keratitis can cause severe ulcers that can also lead to perforation of the eye or permanent scarring with reduced vision after healing. To be truly effective, the treatment must be directed to the etiological agent, for which it is suggested, especially in cases of rapid progression or deep ulcers, to carry out microbiological tests for a correct identification of the causal agent [1,3]. Before the laboratory confirmation it is possible to suspect some infectious forms, such as pseudomonas keratitis and acanthamoeba keratitis [1], but in many cases the clinical picture does not allow to formulate a correct diagnostic hypothesis. The inflammatory response, the similarity of some clinical features and the effect of any recently performed empirical antimicrobial treatments make the etiological evaluation of microbial corneal infections even more difficult. Culture testing of corneal scraping or conjunctival brushing is therefore a necessary study in severe or complex forms and in those that have not responded to empirical or broad-spectrum treatment and is the prerequisite for culture-guided antimicrobial therapy [17]. However, studies of large series of patients with suspected microbial keratitis show that standard culture tests demonstrate the presence of microorganisms in only about 60% of cases [18]. In some cases it is not possible to isolate the etiological agent, because it cannot be identified with common culture tests; in other cases, the effect of previous therapies or of the immune response considerably reduces the microbial load, preventing the isolation of the microorganism culture. In the present study the ability of SEM cytological examination to detect microorganisms not identified with culture tests in suspected microbial keratitis is evaluated. The results indicate a high reliability of the clinical evaluation of suspected microbial origin of the keratitis (62/65 cases) and a high ability of the SEM examination to detect the pathogen, not highlighted with traditional culture methods. The study also shows the frequent discovery of pathogens considered rare, such as Acanthamoeba, Candida, Chlamydia, Mycobacteria and Mycoplasmas (Figure 3), and the possible association of multiple pathogens in corneal infections that do not improve with wide-ranging empirical therapies. In 47.69% of cases a multiple infection was detected, caused by two or more pathogens.

The Authors therefore believe that in these forms of keratitis, even if the usual culture tests are negative, further diagnostic tests should be performed. The reliability of the results obtained is confirmed by the good response to the therapy based on SEM observation and on clinical criteria, even if not targeted to the antibiogram. A further advantage of the cytological examination of the cellular morphology in Scanning Electron Microscopy is the possibility to identify the inflammatory infiltrate: the characteristics of the inflammatory response indicates the pathological potential of the microorganisms found and allows us to suspect other pathogenetic mechanisms, such as allergic etiology, in presence of eosinophils or mast cells. The limitation of the SEM exam consists in the lack of antibiogram or antimycogram to assess the drugs that can be used for topical ophthalmological administration, while these data are usually available in case of isolation of the pathogenfrom plate culture. This limitation implies the need to establish the therapeutic strategy on the basis of the therapies suggested by literature data. Furthermore, the assessment of antimicrobial resistance is an indication of the degree of virulence of the microbe. This information is missing in the cytological evaluation in SEM. Table 2 Summarizes the advantages and limitations of each method.

Finally, the proposed method shows good tolerability, as it does not require corneal scraping, but only of the superior tarsal conjunctiva, which is in close contact with the corneal surface.

## 5. Conclusions

The cytological examination of the cellular morphology in Scanning Electron Microscopy appears useful and effective in detecting the presence of microbial agents in patients with clinical suspicion of ocular surface infection, in which it was not possible to isolate the microorganism with standard culture tests, and that had clinical improvement with broad-spectrum therapies. This method is already used in ophthalmology for assessing the degree of sufferance of the epithelial cells [11] and for identifying pathogenic microorganisms [9,10]. The exam is based on the recognition of the various microbial species based on their size and morphological characteristics. In most of these cases, we identified pathogens that usually do not grow in normal culture media, such as Mycoplasmas, Mycobacteria, Chlamydia and Acanthamoeba. In some cases, the method appeared more sensitive than the culture test in highlighting bacterial or fungal species generally capable of developing in culture media, but whose identification is hindered by the therapies performed or by a low microbial load. The results of the study indicate a high sensitivity (62/65 cases) of the described method, based on the SEM examination, and a good tolerability, linked to the sampling methods. Limitations are need for specific equipment (SEM) and a learning curve to gain sufficient experience in cytological evaluations and in the identification of pathogens; moreover, the proposed method, unlike standard culture tests, does not allow to detect the spectrum of sensitivity to antimicrobials and the minimum inhibitory concentration (MIC) for the various drugs available. The authors suggest to perform this exam in cases of worsening keratitis of suspected microbial origin, in which the pathogen could not be identified with standard tests, and that have not responded to broad spectrum-antibiotic therapies Further studies are necessary to define the correct indications for the execution of the examination, to standardize the timing of the follow-up in the various pathological conditions and to improve its diffusion with digitized systems that facilitate the identification of the microorganisms.

## Figures and Tables

**Table 1:** Clinical data of the examined patients.

ID Patient	Age/ Sex	Systemic Pathologies	Ocular Pathology	Microorganisms	Therapy	Resolution Time
1	42/m	Hypertension	Keratoconjunctivitis	Candida Mycobacterium	Fluconazole 0.2% Chlortetracycline 1%	21 days
2	37/f	--	Keratitis	Mycoplasma Aspergillus	Chlortetracycline Fluconazole Povidone-iodine	21 days
3	47/m	--	Keratoconjunctivitis	Candida Acanthamoeba	Chlorhexidine 0.2% Fluconazole 0.2% PHMB 0.2%	35 days
4	59/m	Hypertension	Keratoconjunctivitis	None (Epith.metaplasia)	Hydrocortisone Ialuronic Acid	28 days
5	48/f	--	Keratoconjunctivitis	Micrococci	Chlortetracycline	21 days
6	64/f	Hypertension Dyslipidemia	Keratoconjunctivitis	Mycoplasma Pseudomonas	Chlortetracycline Levofloxacin	21 days
7	61/f	Dysthyroidism	Keratoconjunctivitis	Candida	Fluconazole	21 days
8	18/m	Hypertension Dyslipidemia Cardiopathy	Keratoconjunctivitis	Mycoplasma Chlamydia Criptococcus	Chlortetracycline Fluconazole Chlorhexidine 0.2%	35 days
9	55/f	Cancer	Keratoconjunctivitis	Cocci	Gentamicin Ciprofloxacin	14 days
10	56/m	Psychosis Dyslipidemia	Keratoconjunctivitis	Mycoplasma Acanthamoeba	Chlortetracycline PHMB 0.2% Chlorhexidine 0.2%	35 days
11	48/m		Keratoconjunctivitis	Mycobacterium	Chlortetracycline Azithromycin	21 days
12	63/m	Diabetes Hypertension	Corneal Ulcer	Acanthamoeba Candida Cocci	PHMB 0.2% Chlorhexidine 0.2% Ciprofloxacin	28 days
13	67/f	Hypertension Dyslipidemia	Keratoconjunctivitis	Mycoplasma	Chlortetracycline Lomefloxacin	21 days
14	60/m	Hypertension	Corneal infiltrates	Mycobacterium Cladosporium	Chlortetracycline Fluconazole	21 days
15	40/m	---	Corneal Ulcer	Acanthamoeba Candida Micrococci	PHMB 0.2% Fluconazole Chlorhexidine 0.2% Levofloxacin	35 days
16	53/f	---	Corneal abscess	Candida	Voriconazole 0.2%	21 days
17	15/f	---	Keratoconjunctivitis	Mycoplasma	Chlortetracycline Levofloxacin	28 days
18	51/m	---	Keratoconjunctivitis	Acanthamoeba HSV I	PHMB 0.2% Chlorhexidine 0.2% Acyclovir	42 days
19	71/f	Diabetes	Keratoconjunctivitis	Micrococci	Tetracycline	21 days
20	29/m	Prostatitis	Keratitis	Candida Acanthamoeba	Fluconazole PHMB 0.2% Chlorhexidine 0.2%	28 days
21	75/f	Hypertension Cardiopathy Dyslipidemia Diabetes	Keratitis	Acanthamoeba	PHMB 0.2% Chlorhexidine 0.2% Chloramphenicol	42 days
22	69/f	Hypertension Cardiopathy Diabetes	Keratitis	Mycoplasma	Chlortetracycline	35 days
23	40/f	---	Keratoconjunctivitis	HSVI	Acyclovir Chlorhexidine 0.2%	14 days
24	30/f	---	Keratoconjunctivitis	Mycoplasma Acanthamoeba	Chlortetracycline PHMB 0.2% Chlorhexidine 0.2%	35 days
25	41/f	---	Keratitis	Cocci	Chloramphenicol Chlortetracycline	10 days
26	25/m	Hypertension Cardiopathy	Keratoconjunctivitis	Mycobacterium	Chlortetracycline Chlorhexidine 0.2%	28 dys
27	60/m	Hypertension Diabetes	Keratoconjunctivitis	Candida Acanthamoeba	Fluconazole PHMB 0.2% Chlorhexidine 0.2%	35 days
28	66/f	Hypertension Diabetes	Keratoconjunctivitis	Acanthamoeba Chlamydia	PHMB 0.2% Chlorhexidine 0.2% Chlortetracycline	35 days
29	60/m	Hypertension Diabetes	Keratoconjunctivitis	Candida	Fluconazole	28 days
30	44/m	--	Keratoconjunctivitis	Chlamydia Mycoplasma	Chlortetracycline Chloramphenicol	21 days
31	58/m	Hypertension Dyslipidemia	Keratoconjunctivitis	Candida Micobacterium	Fluconazole Chlortetracycline Ofloxacin	28 days
32	39/m	---	Keratoconjunctivitis	None (Eosinofils)	Ketotifen Hydrocortisone Ialuronic Acid	14 days
33	60/m	Hypertension	Keratoconjunctivitis	Candida Acanthamoeba	Fluconazole PHMB 0.2% Chlorhexidine 0.2%	28 days
34	57/m	---	Keratoconjunctivitis	Acanthamoeba Micobacterium	PHMB 0.2% Chlorhexidine 0.2% Chlortetracycline	35 days
35	62/m	---	Keratoconjunctivitis	Mycoplasma Pseudomonas	Levofloxacin Chlorhexidine 0.2% Chlortetracycline	21 days
36	73/f	Hypertension Diabetes	Keratitis	Candida	Fluconazole Chlorhexidine	32 days
37	52/f	Hypothyroidism	Keratoconjunctivitis	Chlamydia HSV II	Chlortetracycline Acyclovir, Ofloxacin	28 days
38	43/f	Polycystic ovary	Keratoconjunctivitis	Candida	Fluconazole Chlortetracycline	21 days
39	66/m	Hypertension	Keratoconjunctivitis	Candida Acanthamoeba	FluconazolePHMB 0.2% Chlorhexidine 0.2%	42 days
40	35/m	---	Keratoconjunctivitis	Mycobacterium	Chlortetracycline Sulfamethoxazole	21 days
41	60/f	---	Keratoconjunctivitis	Aspergillus fumigatus	Fluconazole Povidone-iodine (PVP-I)	28 days
42	56/f	Dysthyroidism	Keratoconjunctivitis	Mycobacterium NTN	Chlortetracycline Povidone-iodine (PVP-I)	21 days
43	55/f	---	Keratitis	Acanthamoeba	PHMB 0.2% Chlorhexidine 0.2%	35 days
44	52/m	---	Keratoconjunctivitis	Mycoplasma Acanthamoeba	Chlortetracycline PHMB 0.2% Chlorhexidine 0.2%	28 days
45	70/f	Hypertension Dyslipidemia Cardiopathy	Corneal Infiltrates	Candida Acanthamoeba Micrococci	Fluconazole PHMB 0.2% Chlorhexidine 0.2% Ciprofloxacin	49 days
46	67/f	Hypertension	Keratitis punctata	None (Globet cells deficit)	Ialuronic Acid Hydrocortisone Carbopol gel	14 days
47	2/f	Hypertension	Keratoconjunctivitis	Candida	Fluconazole Chlorhexidine 0.2%	28 days
48	61/f		Keratoconjunctivitis	Acanthamoeba Mycoplasma	PHMB 0.2% Chlorhexidine 0.2% Chlortetracycline Chloramphenicol	35 days
49	42/m	---	Keratoconjunctivitis	Mycoplasma Cocci	Chlortetracycline Levofloxacin	21 days
50	34/f	---	Keratoconjunctivitis	Acanthamoeba Candida	PHMB 0.2% Chlorhexidine 0.2% Fluconazole	35 days
51	69/f	Psoriasis	Keratoconjunctivitis	Acanthamoeba Candida	PHMB 0.2% Chlorhexidine 0.2% Fluconazole	35 days
52	53/f	---	Keratoconjunctivitis	Mycoplasma Candida Chlamydia	Chlortetracycline Fluconazole Azithromycin	21 days
53	64/f	Hypertension Cardiopathy	Keratoconjunctivitis	Mycobacterium	Chlortetracycline Cloramphenicol	21 days
54	66/m	Hypertension	Keratoconjunctivitis	Pseudomonas	Gentamicin Lomefloxacin	14 days
55	63/f	Hypertension Dyslipidemia	Keratoconjunctivitis	Mycoplasma Aspergillus	Chlortetracycline Fluconazole Chlorhexidine 0.2%	28 days
56	56/f	Hypertension Diabetes Dyslipidemia	Keractoconjunctivitis	Acanthamoeba	PHMB 0.2% Chlorhexidine 0.2%	35 days
57	75/f	Hypertension	Keratitis	Candida	Fluconazole Povidone-iodine (PVP-I)	28 days
58	66/f	Hypertension	Keratoconjunctivitis	Chlamydia	Chlortetracycline Azithromycin	21 days
59	62/m	Cancer	Keratoconjunctivitis	Acanthamoeba	PHMB 0.2% Chlorhexidine 0.2% Chlortetracycline	28 days
60	86/f	Dysthyroidism Parkinson disease	Keratoconjunctivitis	Mycoplasma Chlamydia	Chlortetracycline Lomefloxacin	28 days
61	54/m	Hypertension	Keratoconjunctivitis	Candida Mycoplasma	Fluconazole Chlortetracycline Chlorhexidine 0.2%	21 days
62	33/m	---	Keratitis punctata	None (Eosinophils)	Olopatadine Hyaluronic acid Spaglumic Acid	18 days
63	43/m	Hypertension	Keratoconjunctivitis	Chlamydia	Chlortetracycline Azytromicin	28 days
64	35/f	---	Keratoconjunctivitis	Mycoplasma	Chlortetracycline Spaglumic Acid	21 days
65	38/m	Polycystic ovary	Keratoconjunctivitis	Candida Acanthamoeba	Fluconazole PHMB 0.2% Chlorhexidine 0.2%	35 days

**Table 2:** Advantages and limitations of SEM examination and Microbiologic standard cultures.

Sem Examination		Microbiologic Cultures	
Advantages	Disadvantages	Advantages	Disadvantages
Simple conjunctival collection Rapid analysis (48–72 h) High specificity and sensitivity Wide range of indications: allergic and autoimmune diseases; bacteria, mycetes and protozoa infections Evaluation of WBC population present	Operator-dependent technique Viral agents are not directly detected Need for suitable equipment (SEM)	Simple conjunctival sample collection Relatively quick times for bacteria (about 3 days) and mycetes (7 days) isolation High specificity and sensitivity Possibility of carrying out antibiogram and antimicogram	Operator-dependent technique Viral agents are not directly highlighted No allergic and autoimmune diseases are detected The results depend on the microbiological cultures used Some pathogenic agents are detected only after a specific request and in long times

## Data Availability

The data presented in this study are available upon request of the corresponding author. The data are not publicly available as they are elements obtained from direct study on biological samples provided by the patient and treated by the doctor. These patients have freely subscribed to participation in this study for the supply of biological material.
